# Improvement of Esterifying Power of Isolated *Bacillus velezensis* from Daqu by Atmospheric Pressure and Room Temperature Plasma Mutagenesis

**DOI:** 10.3390/foods14050800

**Published:** 2025-02-26

**Authors:** Chuan Song, Tongwei Guan, Zhuang Xiong, Xiaodie Chen, Wenying Tu, Yanping Xu, Xiyue Yan, Qiang Li

**Affiliations:** 1 Postdoctoral Research Station, Luzhou Laojiao Co., Ltd., Luzhou 646000, China; songchuan@lzlj.com; 2 Food Microbiology Key Laboratory of Sichuan Province, College of Food and Biological Engineering, Xihua University, Chengdu 610039, China; 3 Key Laboratory of Coarse Cereal Processing, Ministry of Agriculture and Rural Affairs, Sichuan Engineering & Technology Research Center of Coarse Cereal Industrialization, School of Food and Biological Engineering, Chengdu University, Chengdu 610106, China; xiongzhuang2000@126.com (Z.X.); cxd0512@126.com (X.C.); twy1712076841@126.com (W.T.); xuyp0422@163.com (Y.X.); 18613235090@163.com (X.Y.)

**Keywords:** Baijiu, microorganisms, ARTP mutagenesis, strain breeding, fermentation

## Abstract

Strong-flavor Baijiu, a widely popular distilled spirit in China, derives its characteristic aroma and quality largely from ethyl hexanoate, a key flavor compound. The concentration of ethyl hexanoate, influenced by its precursor hexanoic acid, is critical in defining the style and quality of this Baijiu variety. In this study, atmospheric and room temperature plasma (ARTP) mutagenesis technology was applied to strains isolated from Strong-flavor Daqu to enhance their acid and ester production capabilities. A hexanoic acid-producing strain, identified as *Bacillus velezensis* WY4 through morphological, physiological, biochemical, and molecular analyses, was used as the starting strain. Following 90 s of ARTP exposure, a mutant strain, WY4-3, was successfully developed, achieving a balance between high mutation diversity and moderate lethality. WY4-3 exhibited robust growth across a pH range of 4.2 to 5.0 and demonstrated high ethanol tolerance. After five days of fermentation, WY4-3 produced 0.36 g/L of total acid and 0.528 g/L of total ester, surpassing the wild-type strain. Enzymatic activity assays revealed significant enhancements in amylase (9.13%), saccharifying enzyme (101.72%), and esterification (573.71%) activities in WY4-3. Validation in multiple artificial esterification systems further confirmed the superior ester production capacity of this mutant strain. These findings enrich the microbial germplasm resources for Baijiu brewing and provide a solid foundation for strain selection and genetic improvement in Baijiu production processes. This study highlights the potential of ARTP mutagenesis in optimizing brewing microorganisms and improving the quality and consistency of Strong-flavor Baijiu.

## 1. Introduction

Chinese Baijiu, as a significant member of the global distilled spirits family, holds a prominent position in the global alcoholic beverage market due to its unique brewing process and distinctive flavor characteristics [1]. Among its varieties, Strong-flavor Baijiu is particularly favored by consumers for its rich aroma, smooth taste, and distinctive style, accounting for over 50% of the sales in the Chinese Baijiu market [2]. The quality and flavor of Strong-flavor Baijiu are primarily derived from its complex microbial fermentation process, especially the microbial community in Daqu. These microbes contribute to Baijiu’s unique flavor profile through saccharification, fermentation, and aroma generation [3,4,5]. Ethyl hexanoate, the primary flavor compound of Strong-flavor Baijiu, plays a decisive role in determining its quality and taste, while its precursor, hexanoic acid, directly influences the production of ethyl hexanoate [6,7]. Therefore, an in-depth study of the microbial community in Strong-flavor Daqu, particularly the acid- and ester-producing microbes, is crucial for enhancing Baijiu quality and optimizing brewing processes.

In recent years, rapid advancements in microbiology and fermentation engineering have led to significant progress in Baijiu brewing research. Traditionally, the fermentation of Strong-flavor Baijiu relies heavily on the naturally occurring microbial community, which produces a rich array of flavor compounds through complex interactions during the brewing process [8,9]. However, the types and numbers of microbes involved in natural fermentation are difficult to control, leading to inconsistencies in Baijiu quality and flavor [10]. To address this challenge, researchers have explored the application of modern biotechnological methods to isolate, identify, and improve the microorganisms involved in Baijiu brewing [11,12]. Using morphological observations, physiological and biochemical reactions, and molecular biology techniques, researchers have successfully isolated various acid- and ester-producing microorganisms from Strong-flavor Daqu, such as *Clostridium*, *Ruminococcaceae*, *Megasphaera*, and *Bacillus* [13,14,15,16]. These findings have enriched the microbial resources available for Baijiu brewing [17].

In terms of microbial improvement, mutagenesis breeding has gained widespread attention due to its efficiency and rapidity. Atmospheric and room temperature plasma (ARTP) technology, an innovative mutagenesis method, offers high mutation rates, good genetic stability, operational simplicity, and eco-friendliness, demonstrating significant potential in microbial breeding [18,19,20]. Studies have shown that ARTP mutagenesis can significantly enhance microbial metabolic activity, product yield, and stress resistance, providing a new approach for improving Baijiu brewing microorganisms.

During the long fermentation process of Baijiu production, the nutrients in pit mud are gradually depleted or imbalanced, while harmful substances like ferrous lactate and calcium lactate accumulate, and the number of beneficial functional microorganisms declines. The effective maintenance of pit mud quality is essential for sustaining Baijiu production [21]. Hexanoic acid-producing microorganisms are critical functional microbes in Baijiu brewing, playing a key role in the formation of the main aroma compound, ethyl hexanoate, in Strong-flavor Baijiu. Studies have shown that these microbes regulate the maintenance of old fermentation pits, and pit mud enriched with high-performing hexanoic acid-producing microorganisms yields Strong-flavor Baijiu with high ethyl hexanoate content, delicate aroma, and clean, smooth taste.

Previous production practices have demonstrated that adding microbial agents enriched with hexanoic acid-producing microbes to pit mud significantly increases the number of functional microorganisms, ethyl hexanoate content in Baijiu, and the percentage of premium-grade products [13]. However, naturally isolated hexanoic acid-producing microbes often exhibit low and unstable acid production efficiencies. Moreover, studies on the genetic improvement of fermentation strains for Strong-flavor Baijiu remain scarce [16]. Traditional mutagenesis breeding methods, which suffer from low mutation rates and poor genetic stability, are insufficient to meet the modern Baijiu industry’s demand for high-yield and high-quality strains [9].

This study aimed to use ARTP mutagenesis technology to improve acid- and ester-producing microorganisms isolated from Strong-flavor Daqu. The goal was to obtain mutant strains with enhanced acid and ester production capabilities, thereby providing high-quality microbial resources for Baijiu brewing.

## 2. Materials and Methods

### 2.1. Isolation of Strains from Daqu

The test strains were isolated from Strong-flavor Baijiu Daqu provided by Luzhou Laojiao Co., Ltd., Luzhou, China. A 1 g sample of Strong-flavor Baijiu Daqu was dissolved in 100 mL of sterile distilled water. In the pre-experiment, we found that the use of sterile distilled water had no significant effect on the survival of the WY4 strain. Because sodium ions in saline may affect the osmotic equilibrium of the subsequent medium, we used sterile distilled water for serial dilutions to concentrations of 10^−3^ to 10^−5^ g/L. Because of the limited absorptive capacity of the agar surface, we applied 1 mL of supernatant to the ethanol-sodium acetate intermediate (composition: yeast extract, 1.0 g; sodium acetate, 0.5 g; MgSO_4_·7H_2_O, 0.02 g; K_2_HPO_4_, 0.04 g; (NH_4_)_2_SO_4_, 0.05 g; agar, 2 g; anhydrous ethanol, 2 mL; water, 100 mL). The plates were incubated at 32 °C for 1–2 days, and purified isolates were obtained using the streak plate method [22]. All medium components and reagents (e.g., yeast extract and agar) were purchased from Sigma-Aldrich (St. Louis, MO, USA).

### 2.2. Acid and Gas Production Assays

The acid and gas production potentials of the isolated pure cultures were assessed. The acid production potential of the isolates was determined using the copper sulfate colorimetric method [23]. Each isolate was inoculated into an ethanol-sodium acetate liquid medium and incubated at 32 °C for 5 days in a constant-temperature incubator (BJPX-H30L, BIOBASE, Jinan, China). Subsequently, 4 mL of the fermentation broth was transferred to a test tube, followed by the addition of 1 mL of 2% copper sulfate solution and 1 mL of ether. The mixture was shaken thoroughly and allowed to stand for 30 min before use. The color of the ether layer was then observed. A bluish-green color with precipitation indicated acid production in the fermentation broth, with a deeper coloration corresponding to higher acid concentrations. Gas production was evaluated using a Durham tube [24]. The Durham tube was then submerged in the medium (yeast extract: 1.0 g/L, sodium acetate: 0.5 g/L, MgSO_4_·7H_2_O: 0.02 g/L, K_2_HPO_4_: 0.04 g/L, (NH_4_)_2_SO_4_: 0.05 g/L, and anhydrous ethanol: 2% *v*/*v*). After sterilization, each isolate was inoculated into the medium and placed in a shaker (ZQZY-AS9, Shanghai Zhichu Instrument Co., Ltd., Shanghai, China) for 3 days at 32 °C with shaking at 100 rpm. Gas production was confirmed by the presence of bubbles in the Durham tube.

### 2.3. Colony Morphology Observation, Growth Curve Determination, and Evolutionary Analysis

A purified strain with potential acid and gas production capabilities was successfully isolated and designated WY4. WY4 was inoculated onto an ethanol-sodium acetate solid medium and incubated at 32 °C under inverted conditions for 3 days. The morphological characteristics of the colonies were observed and recorded, and Gram staining was performed to determine cell color and morphology.

To assess the growth curve, 1 mL of the WY4 strain was inoculated into 100 mL of ethanol-sodium acetate liquid medium and cultured at 32 °C with shaking at 120 rpm. Samples were collected every 24 h, and the optical density (OD) at 600 nm was measured using a UV-1800 spectrophotometer (Shimadzu, Kyoto, Japan). The uninoculated medium served as a blank control. A growth curve was plotted using the culture time as the *x*-axis and OD_600_ as the *y*-axis.

Bacterial cells from the fermentation broth were collected, and total genomic DNA of WY4 was extracted using the OMEGA Bacterial Genomic DNA Extraction Kit (Omega Bio-Tek, Norcross, GA, USA). The 16S rRNA gene of the WY4 strain was amplified using universal primers 27F (5′-AGAGTTTGATCCTGGCTCAG-3′) and 1492R (5′-ACGGTTACCTTGTTACGACTT-3′). The PCR reaction system (QuantStudio5, Thermo Fisher Scientific, Waltham, MA, USA) and amplification conditions were as previously reported [25]. The PCR products were verified by agarose gel electrophoresis and subsequently sequenced by Beijing Tsingke Biotech Co., Ltd. (Beijing, China). The obtained sequences were compared using the Basic Local Alignment Search Tool (BLAST v2.16.0, https://blast.ncbi.nlm.nih.gov/Blast.cgi, accessed on 10 March 2023) to identify closely related species, and a phylogenetic tree was constructed using the neighbor-joining method with reference sequences.

### 2.4. ARTP Mutagenesis of Isolated Strains

The isolated strain was subjected to mutagenesis using the ARTP mutagenesis system (ARTP-II, TMAXTREE Biotechnology Co., Ltd., Wuxi, China). First, the ARTP mutagenesis slide was sterilized for 30 min. Subsequently, 10 μL of the bacterial suspension was evenly spread onto the surface of the slide. The slides were exposed to mutagenesis treatments for 0, 30, 60, 90, 120, and 150 s intervals. After mutagenesis, the slides were placed in sterilized EP tubes containing culture medium, vortexed for 1 min, and removed from the tubes. Next, 10 μL of the mutagenized bacterial suspension was spread on an ethanol-sodium acetate solid medium. Each treatment was performed in three biological replicates. The plates were incubated at 32 °C for 72 h until colonies appeared. The lethality rate of the strains was carefully observed and calculated. A lethality curve was plotted with mutagenesis duration on the *x*-axis and lethality rate on the *y*-axis to determine the optimal ARTP treatment time.

The lethality rate was calculated using the following formula: Lethality rate = (Q — Q_1_)/Q, where Q represents the total number of colonies in the control group and Q_1_ represents the total number of colonies after mutagenesis.

### 2.5. Screening of Mutagenized Strains

The strain WY4 was subjected to mutagenesis using the optimal ARTP treatment time. The mutagenized bacterial suspension was serially diluted to 10^−1^ to 10^−3^, and 100 μL of each dilution was spread onto ethanol-sodium acetate solid medium plates. After incubation at 32 °C for 48 h, single colonies were isolated and purified using the streak plate method. Purified colonies were then inoculated into ethanol-sodium acetate liquid medium and cultured at 32 °C with shaking at 120 rpm for 5 days.

The total acid and ester contents were measured according to the national standard GB/T 10781.1-2021 [26], which is widely adopted for Baijiu quality control. The total acid content was quantified via titration with 0.1 mol/L NaOH, using phenolphthalein as an indicator. The total ester content was determined by saponification with 0.1 mol/L NaOH, followed by back titration with 0.1 mol/L H_2_SO_4_, as described in the GB/T 10781.1-2021 [26]. Strains with high acid and ester production were selected for preservation (mutant strains were preserved in 20% glycerol at −80 °C). Subsequently, the growth curves of the mutagenized strains were analyzed using the method described earlier.

### 2.6. Fermentation Condition Optimization for Mutagenized Strains

To optimize the fermentation conditions for mutagenized strains, several key parameters were evaluated. The initial pH of the ethanol-sodium acetate liquid medium was adjusted to 4.2, 4.4, 4.6, 4.8, and 5.0 using lactic acid solution. Both mutagenized and non-mutagenized strains (100 μL) were inoculated into the respective media and cultured at 32 °C with shaking at 120 rpm. The OD of the fermentation broth was measured at 12, 24, 36, 48, and 50 h to determine the optimal pH for fermentation.

To optimize the ethanol concentration, anhydrous ethanol was added to the ethanol-sodium acetate medium at final concentrations of 1%, 2%, 3%, 4%, and 5%. Mutagenized and non-mutagenized strains (100 μL) were inoculated into the medium and cultured under the same conditions as described above. OD values were measured at the same intervals to identify the optimal ethanol concentrations for each strain.

Fermentation temperature optimization was performed by inoculating 100 μL of each strain into ethanol-sodium acetate liquid medium and incubating at 24 °C, 28 °C, 32 °C, 36 °C, and 40 °C in constant-temperature incubators with shaking at 120 rpm. OD values were recorded at the same time points to determine the most suitable fermentation temperature for each strain.

The effects of glucose and NaCl concentrations on fermentation performance were evaluated by adding glucose or NaCl to ethanol-sodium acetate medium at final concentrations of 5, 10, 15, 20, and 25 g/100 mL. Both strains were inoculated and cultured under the same conditions, and the OD was measured at the same intervals. The results were used to determine the optimal glucose and NaCl concentrations.

Finally, mutagenized, and non-mutagenized strains were inoculated into ethanol-sodium acetate liquid medium and fermented under the optimal conditions determined above. The total acid and ester contents in the fermentation broth were measured after 1, 2, 3, 4, and 5 days of fermentation. Each treatment was performed in triplicate.

### 2.7. Enzyme Activity and Stability Assays

Sorghum grains were ground and sieved using a 100-mesh screen. A 50 g sample of sorghum powder was accurately weighed into an Erlenmeyer flask (Thermo Fisher Scientific, Waltham, MA, USA), sterilized, and mixed with 150 mL of sterile distilled water. The inoculum density was adjusted to 1 × 10⁶ CFU/mL using a hemocytometer (Marienfeld Superior^®^, Lauda-Königshofen, Germany). Subsequently, 5 mL of log-phase cultures of both mutagenized and non-mutagenized WY4 strains were inoculated into the flasks. The mixture was incubated at 32 °C with shaking at 120 rpm in a constant-temperature shaking incubator for 5 days. After fermentation, the mixture was air-dried at 40 °C for 48 h in a ventilated drying oven (Memmert UF260, MEMMERT (Shanghai) Trading Co., Ltd., Shanghai, China).

For enzyme extraction, 10 g of the fermented powder was accurately weighed and placed in a 100 mL beaker with 50 mL of phosphate buffer (pH 7.4). The mixture was stirred until it was uniform and incubated in a 40 °C water bath, with stirring every 5 min. After 60 min, the extract was filtered to obtain a crude enzyme solution. The activities of amylase, saccharifying enzyme, and protease in the crude enzyme solution were measured as previously described methods (Amylase activity was measured using the DNS method, which detects reducing sugars released from starch hydrolysis. Saccharifying enzyme activity was determined using the GOPOD assay, which quantifies glucose oxidation. Protease activity was assessed using the Folin-Ciocalteu method, which measures tyrosine release from casein hydrolysis) [27,28,29].

To evaluate the esterification activity, 1 mL of hexanoic acid was accurately transferred to a 100 mL volumetric flask and diluted to the mark with 20% ethanol. Then, 5 g of fermented sorghum powder was added to a distillation flask, along with 100 mL of the hexanoic acid-ethanol mixture. The mixture was incubated at 32 °C for 100 h to allow for esterification. After incubation, 50 mL of pure water was added, and the mixture was distilled to obtain 100 mL of distillate. Non-enzymatic esterification was confirmed to be negligible under the experimental conditions based on control experiments without microbial inoculation, as previously reported by Chen et al. [30]. The content of ethyl hexanoate in the distillate was measured to calculate the esterification activity of the strains before and after mutagenesis.

To analyze genetic stability, the mutagenized strain WY4-1 was cultured in a liquid medium until the logarithmic phase and then serially transferred for six consecutive generations. At each generation, the culture was subjected to fermentation, and the esterification activity of the strain was determined. The results were used to evaluate the genetic stability of the mutagenized strain.

### 2.8. Evaluation of Mutagenized Strain Performance in Artificial Esterification Systems

An artificial esterification reaction system was constructed by mixing 5 g of fermented sorghum powder with 100 mL of a mixed acid-alcohol aqueous solution (containing hexanoic acid, lactic acid, acetic acid, butyric acid, and anhydrous ethanol) in a 150 mL Erlenmeyer flask. The reaction mixture was incubated at 35 °C for 100 h to allow for esterification. After incubation, the liquid was distilled, and the distillate was stored at 4 °C. The pH, alcohol content, total acid, and total ester content of the distillate were measured [30,31,32,33,34]. The composition of the acid-alcohol mixture was prepared according to the proportions listed in Table 1.

### 2.9. Statistical Analysis

All experiments were performed in triplicate, and the data are expressed as the mean ± standard deviation (SD). Statistical analysis was performed using one-way ANOVA, followed by Tukey’s post-hoc test (*p* < 0.05) to determine significant differences between groups. All statistical analyses were performed using SPSS 26.0 (IBM, Armonk, NY, USA).

## 3. Results and Analysis

### 3.1. Isolation of Strains and Screening for Potential Hexanoic Acid-Producing Strains

**Figure 1:**
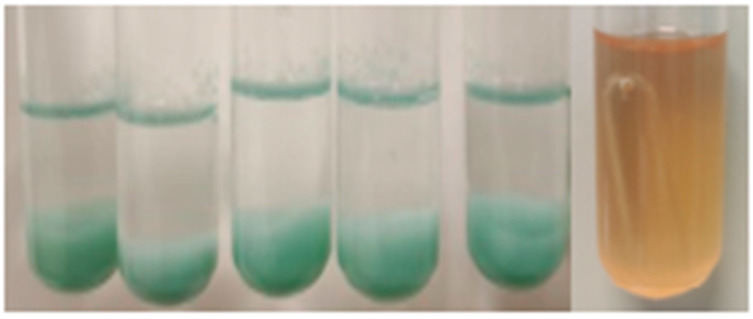
Acid (**left**) and gas (**right**) production by the isolated bacterial strain WY4.

A total of 56 pure bacterial strains with distinct colony morphologies were successfully isolated from Daqu (Luzhou Laojiao Co., Ltd., Luzhou, China). To evaluate their potential for hexanoic acid production, the acid and gas production capacities of these strains were tested using the copper sulfate colorimetric method and the Durham tube assay, respectively. Gas production, primarily CO_2_, is a common byproduct of microbial fermentation and serves as an indirect indicator of the metabolic activity. In the context of Baijiu fermentation, gas production is closely linked to the synthesis of organic acids, including hexanoic acid, which is a precursor of ethyl hexanoate, a key flavor compound in Strong-flavor Baijiu. Therefore, gas production was measured to identify strains with high metabolic activity and the potential for acid and ester production. Among the isolates, strain WY4 produced a light green precipitate and exhibited gas production capability (Figure 1), indicating its potential for hexanoic acid generation. Therefore, strain WY4 was selected as the starting strain for subsequent experiments.

### 3.2. Identification and Growth Curve Determination of Strain WY4

**Figure 2:**
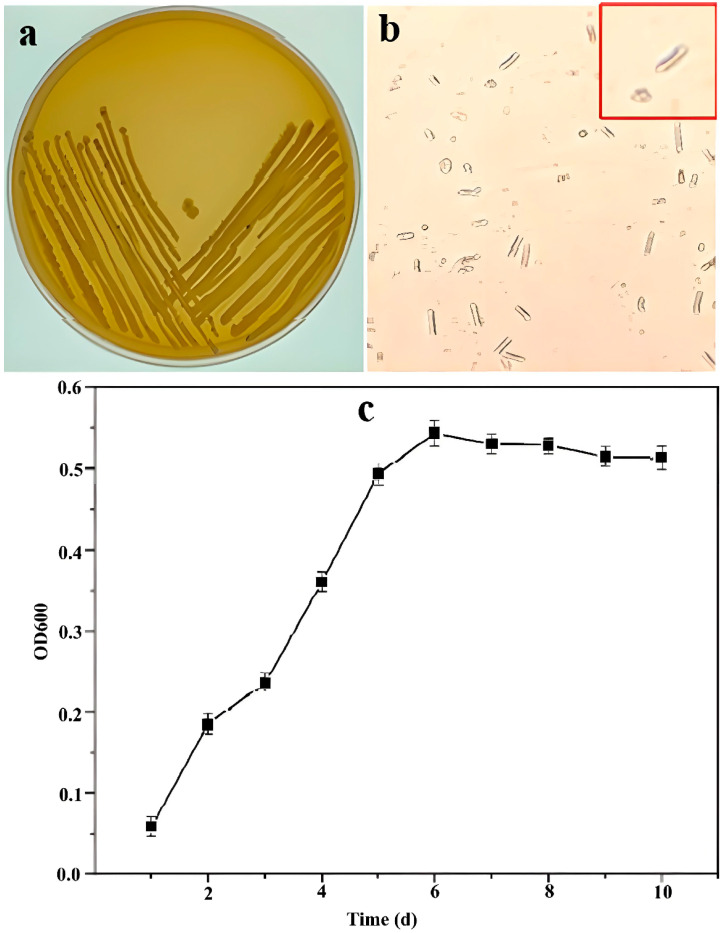
Colony morphology (**a**), Gram staining microscopy observation (**b**), and growth curve (**c**) of the WY4 strain. The red box in (**b**) represents the enlargement of the bacterial cells.

Morphological identification and Gram staining were performed to characterize the isolated strain, WY4. The colonies of WY4 were observed to be milky-white to pale yellow, smooth, opaque, and moist, with a texture that made them difficult to pick up (Figure 2a). Gram staining showed that WY4 was Gram-positive with rod-shaped cells (Figure 2b).

**Figure 3:**
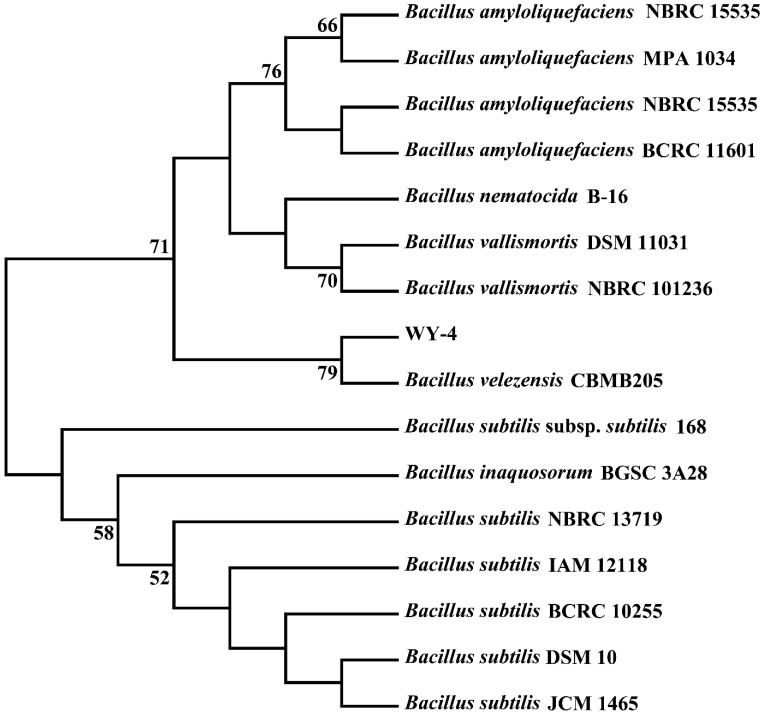
Phylogenetic tree constructed using the Neighbor-Joining method based on the 16S rRNA sequence of the WY4 strain.

The 16S rRNA sequence of WY4, obtained through PCR amplification, was compared using the BLAST tool on the NCBI. The results revealed the highest similarity (99%) to *Bacillus velezensis* CBMB205. Homologous sequences with over 98% similarity were selected to construct a phylogenetic tree using MEGA 7.0 software. Phylogenetic analysis indicated that WY4 is closely related to *Bacillus velezensis* CBMB205, leading to its identification as *Bacillus velezensis* (Figure 3).

The growth curve of WY4 was determined and is shown in Figure 2c. WY4 entered the logarithmic growth phase approximately 1 day after inoculation and transitioned to the stationary phase after 6 days. Therefore, cells in the logarithmic growth phase were used for subsequent experiments involving *Bacillus velezensis* WY4.

### 3.3. ARTP Mutagenesis and Screening of High Acid- and Ester-Producing Strains

**Figure 4:**
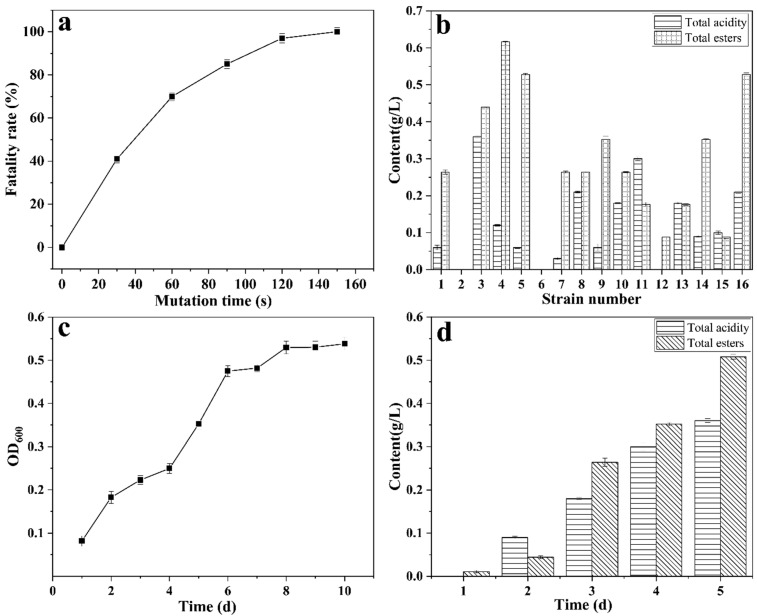
Screening for mutagenic bacteria. (**a**), the trend of the mortality rate of mutated bacteria with the change in mutagenesis time; (**b**) total acid and ester production of 16 selected mutant strains; (**c**) the growth curve of the screened mutant strain WY4-3; (**d**) the total acid and ester content of the screened mutant strain WY4-3 varies with fermentation time (mean ± SD, *n* = 3).

*Bacillus velezensis* WY4 was cultured to the logarithmic growth phase and prepared as a bacterial suspension. The lethality rate of the strain after ARTP mutagenesis at different exposure times was determined. As shown in Figure 4a, the lethality rate was below 50% when the exposure time did not exceed 30 s. At 60 s, the lethality rate increased to 70%, and it further increased to 85% at 90 s (*p* < 0.05). When the exposure time reached 120 s, the lethality rate was 97%. To balance the strain diversity and lethality rate, a mutagenesis duration of 90 s was selected for ARTP mutagenesis of strain WY4.

After 90 s of mutagenesis, single-colony strains were obtained through repeated purification using the streak plate method. Sixteen single-colony strains were selected for fermentation experiments to measure their total acid and total ester production (Figure 4b). Among them, strain 3 exhibited the highest combined production of total acid and total ester, with total acid contents reaching 0.36 g/L and total ester contents reaching 0.528 g/L after 5 days of fermentation (*p* < 0.05). Consequently, strain 3 was designated as the final mutagenized strain, WY4-3.

The growth curve of the mutagenized strain, *Bacillus velezensis* WY4-3, was also determined. The results showed that WY4-3 exhibited slow growth during the initial phase and entered the logarithmic growth phase after 4 days. The growth rate slowed down after 6 days, and the strain reached the stationary phase at 8 days (Figure 4c).

### 3.4. Optimization of Fermentation Conditions for the Mutagenized Strain

From a previous mutagenesis screening, a highly acid- and ester-producing strain, WY4-3, was identified. The fermentation performance of WY4-3 over time is shown in Figure 4d. As fermentation progressed, both the total acid and total ester contents increased. After 5 days of fermentation, the total acid content reached 0.36 g/L, and the total ester content reached 0.508 g/L (*p* < 0.05).

**Figure 5:**
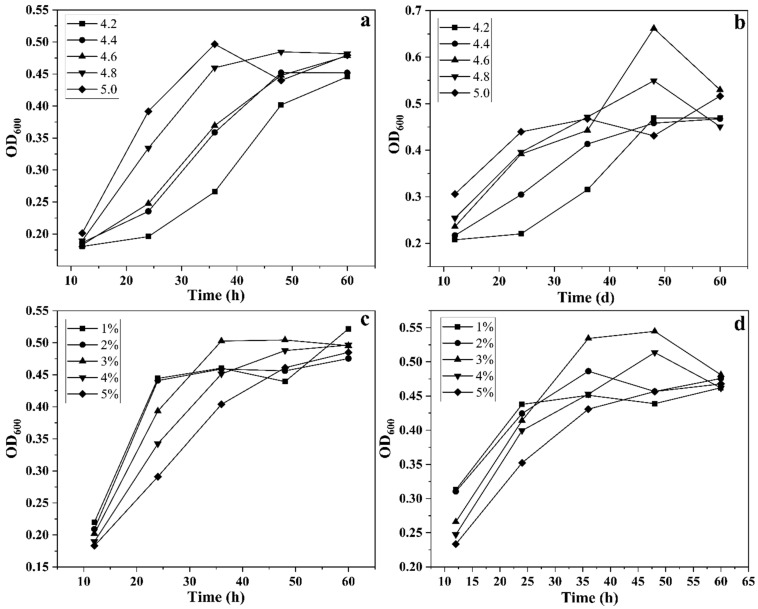
Growth curves of wild-type strain WY4 (**a**,**c**) and mutant strain WY4-3 (**b**,**d**) at different pH values and alcohol concentrations (mean ± SD, n = 3).

**Figure 6:**
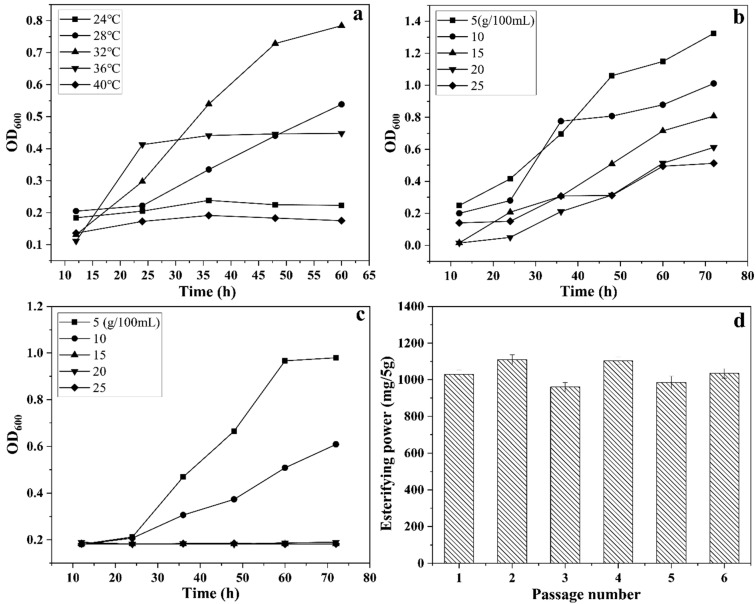
The growth curves of mutagenic bacteria at different temperatures (**a**), different glucose concentrations (**b**), different sodium chloride concentrations (**c**), and the changes in esterifying power under different generations (**d**) (mean ± SD, n = 3).

As shown in Figure 5a,b, *Bacillus velezensis* WY4-3 exhibited growth across a pH range of 4.2 to 5.0. At pH 4.2, the strain demonstrated slower growth during the initial 25 h, but by approximately 60 h of fermentation, the growth of the mutagenized strain was nearly comparable to that of the wild-type strain. Figure 5c,d illustrates the effects of ethanol concentration on growth. Within the ethanol concentration range of 1% to 3%, there was no significant difference in growth between the mutagenized and wild-type strains. However, at ethanol concentrations ≥4%, the mutagenized strain exhibited some inhibition during the first 25 h. After 25 h, the growth levels of the mutagenized and wild-type strains were comparable. Overall, *Bacillus velezensis* demonstrated the ability to grow within an ethanol concentration range of 1% to 5%, indicating that the mutagenized strain retained high ethanol tolerance. As shown in Figure 6a, the optimal growth temperature for the mutagenized strain was 32 °C, under which the biomass increased steadily with incubation time. Growth was significantly inhibited at 40 °C, and growth at 24 °C was also slow. At 28 °C and 36 °C, the growth rate of the strain was relatively lower compared to that at 32 °C.

As shown in Figure 6b, *Bacillus velezensis* WY4-3 grew stably across the range of glucose concentrations tested. However, the overall growth rate of the mutagenized strain decreased as glucose concentration increased. Figure 6c shows the effect of NaCl concentration on growth. The mutagenized strain exhibited good growth at NaCl concentrations of less than 10%. However, when the NaCl concentration exceeded 15%, the growth of the strain ceased entirely.

### 3.5. Changes in Enzyme Activity and Esterification Capacity of Mutagenized Strains

Compared to the non-mutagenized *Bacillus velezensis* WY4, the mutagenized strain WY4-3 exhibited significantly enhanced activities for amylase, saccharifying enzyme, and esterification capacity (*p* < 0.05) (Table 2). Specifically, amylase activity increased by approximately 9.13%, saccharifying enzyme activity increased by 101.72%, and esterification capacity improved by 573.71%. However, the protease activity of the mutagenized strain decreased significantly compared to that of the wild-type strain, with a reduction of approximately 153.79% (*p* < 0.05). To evaluate the genetic stability of the mutagenized strain, WY4-3 was serially subcultured for six generations, and its esterification capacity was measured. As shown in Figure 6d, the synthesis of ethyl hexanoate remained stable at approximately 1110.2 mg/5 g. These results indicate that the mutagenized strain *Bacillus velezensis* exhibits good genetic stability.

### 3.6. Evaluation of Mutagenized Strain Performance in an Artificial Esterification System

**Figure 7:**
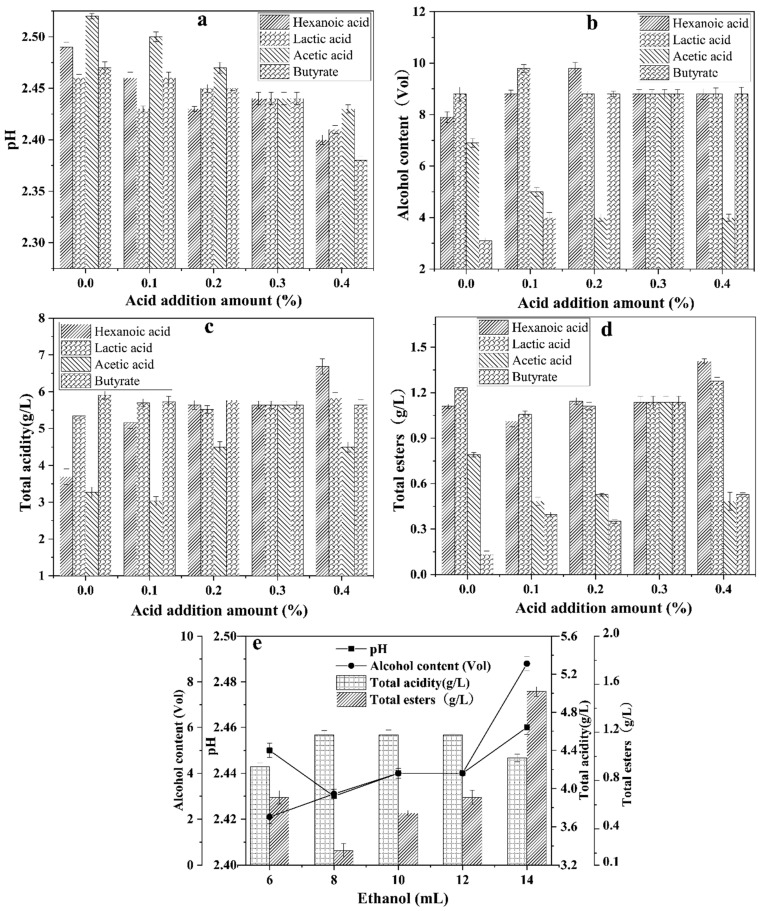
Changes in pH (**a**), alcohol content (**b**), total acid (**c**), and total ester (**d**) of WY4-3 strain induced by artificial esterification system with the addition of acid and ethanol (**e**) (mean ± SD, n = 3).

As shown in Figure 7a, the pH of the artificial esterification system generally decreased with increasing concentrations of hexanoic acid, lactic acid, acetic acid, and butyric acid. However, when 0.3% hexanoic acid and 0.1% lactic acid were added, the pH increased, suggesting that the mutagenized strain WY4-3 of *Bacillus velezensis* consumed hexanoic acid and lactic acid during esterification. From Figure 7b, it can be observed that the alcohol content decreased gradually with increasing acetic acid addition. Conversely, no significant changes were observed in the trends of hexanoic acid, lactic acid, and butyric acid as the alcohol content increased. This indicates that all four acids contributed to the consumption of ethanol to some extent during esterification by the mutagenized strain. As illustrated in Figure 7c, the total acid content showed no significant fluctuations with the addition of lactic acid or butyric acid, indicating that their addition facilitated the generation of total esters during esterification by *Bacillus velezensis*. Figure 7d shows that the total ester content increased with the addition of hexanoic acid, lactic acid, and butyric acid, reaching a maximum of 1.136 g/L when 0.3% of each acid was added. However, the total ester content decreased with the addition of acetic acid, suggesting that the four acids promoted ester generation during the esterification process, albeit with varying efficiencies. Lastly, Figure 7e shows that the pH, total acid, and total ester levels initially decreased and then increased with increasing ethanol concentrations. This indicates that during esterification by *Bacillus velezensis* WY4-3, the rate of acid consumption exceeded the rate of acid production. The dynamic changes in the total ester content could be attributed to the consumption of acids to synthesize esters as the ethanol levels increased. When the system had a high ethanol-to-acid ratio, it prioritized acid synthesis over ester formation.

## 4. Discussion

Strong-flavor Baijiu, a major type of Chinese Baijiu, is widely favored by consumers because of its unique flavor. The formation of these flavor characteristics largely depends on the diversity and activity of microorganisms during the fermentation process [35]. Previous studies have shown that bacteria, fungi, and other microorganisms play critical roles in Baijiu production by not only producing enzymes and flavor compounds but also significantly influencing the fermentation process and the final product’s flavor profile [36].

In this study, we successfully isolated 56 pure strains with distinct colony morphologies from a Strong-flavor Baijiu Daqu. Using copper sulfate colorimetric and Durham tube assays, we identified strain WY4 as having the potential to produce hexanoic acid. This discovery provided an important starting strain for subsequent experiments. Further identification of WY4 revealed it to be *Bacillus velezensis*. The phylogenetic tree constructed based on the 16S rRNA sequence of WY4 (Figure 3) revealed its close relationship with *Bacillus velezensis* CBMB205, with a similarity of 99%. This finding confirms the taxonomic classification of WY4 and supports its potential as a functional microorganism in Baijiu brewing. As a common microorganism in Baijiu brewing, *Bacillus velezensis* has been reported in several studies to possess properties such as amylase, protease, and cellulase production and strong fermentative capabilities [37,38,39]. The growth curve of WY4 was further analyzed, showing that its logarithmic growth phase occurred between 2 and 6 days, consistent with the typical growth characteristics of *Bacillus velezensis*.

ARTP mutagenesis is a rapid and efficient method for microbial genetic improvement and has been widely applied in recent years [40]. Studies have demonstrated that ARTP mutagenesis offers advantages such as a high mutation rate, good genetic stability, and operational simplicity [41]. In this study, we applied ARTP technology to mutagenize the strain WY4 and successfully obtained a high acid- and ester-producing mutant strain, WY4-3. By analyzing the lethality rates at different mutagenesis times, we determined that 90 s of exposure provided an optimal balance between strain diversity and lethality. This result guided the selection of an appropriate mutagenesis duration. Through multiple fermentation assays, we identified WY4-3 as the mutant strain with the highest total acid and ester production, enriching the microbial germplasm resources for Baijiu brewing and offering new possibilities for producing high-ester Baijiu.

To determine the optimal fermentation conditions for WY4-3, we systematically examined its growth and fermentation performance under varying pH values, ethanol concentrations, temperatures, glucose, and NaCl levels. The results showed that WY4-3 could grow across a pH range of 4.2–5.0 and exhibited high ethanol tolerance within a certain concentration range. Additionally, the strain demonstrated optimal growth and fermentation performance at 32 °C. These findings provide crucial process parameters for high-ester Baijiu production. The results also confirmed that WY4-3 has strong environmental adaptability and can thrive under various fermentation conditions.

Enzyme activity and esterification capacity are key indicators for evaluating the performance of brewing microorganisms [42,43]. In this study, we measured the amylase, saccharifying enzyme, and protease activities and esterification capacity of WY4 before and after mutagenesis. The results showed significant increases in amylase and saccharifying enzyme activities and a remarkable 573.71% enhancement in esterification capacity in the mutagenized strain, WY4-3. This indicates that ARTP mutagenesis not only improved the metabolic activity of WY4 but also significantly enhanced its ester production capability. However, a notable decrease of approximately 153.79% was observed in protease activity after mutagenesis, likely due to mutations in genes related to protease synthesis and activity. Nonetheless, given the relatively minor role of protease in Baijiu flavor formation, this change is unlikely to have a substantial impact on the final product’s flavor. These results confirm the effectiveness of ARTP mutagenesis for microbial genetic improvement in Baijiu brewing and provide new insights into the enzymology and metabolism of brewing microorganisms.

To evaluate the ester production capacity of WY4-3 in an artificial esterification system, the strain was inoculated into a mixture of various acids and ethanol. The results showed that WY4-3 exhibited a high-ester production capacity in an artificial system and maintained stable genetic inheritance of this trait. These findings suggest that WY4-3 not only has the potential for high-ester production but also demonstrates good genetic stability.

The mutant strain WY4-3, obtained through ARTP mutagenesis, demonstrated significant improvements in total acid and ester production, making it a promising candidate for industrial applications in Baijiu brewing. The enhanced enzymatic activity and esterification capacity of WY4-3 suggest that it can be used to produce high-ester Baijiu with improved flavor profiles. While the mutagenized strain WY4-3 shows great potential, there are several limitations to consider. The genetic stability of the mutant strain over prolonged fermentation cycles needs to be thoroughly evaluated. Additionally, the environmental adaptability of WY4-3 under varying fermentation conditions must be assessed to ensure its consistent performance. From a safety perspective, it is crucial to confirm that the mutagenesis process does not introduce any harmful traits that could affect human health negatively. Rigorous toxicological assessments and safety evaluations should be conducted before approving this strain for use in food production.

## 5. Conclusions

In this study, we successfully applied ARTP mutagenesis to enhance the acid and ester production capabilities of *Bacillus velezensis* WY4, resulting in the mutant strain WY4-3. The mutant strain exhibited significant improvements in enzymatic activity and esterification capacity, making it a promising candidate for industrial applications in Baijiu brewing. The improved acid and ester production capabilities of WY4-3 could significantly enhance the flavor profile and quality of Strong-flavor Baijiu, leading to increased consumer satisfaction and market competitiveness. Our findings highlight the potential of ARTP mutagenesis in optimizing brewing microorganisms and advancing the quality and consistency of Strong-flavor Baijiu. The strain’s high ethanol tolerance and robust growth under varying fermentation conditions further support its potential for industrial applications. By providing high-quality microbial resources for Baijiu brewing, this study contributes to the sustainable development of the Baijiu industry in China.

Future research should focus on the large-scale application of WY4-3 in Baijiu production, including pilot-scale fermentation trials and long-term stability studies. Additionally, comprehensive safety assessments, including toxicological and allergenicity studies, should be conducted to ensure the strain’s suitability for industrial use.

## Figures and Tables

**Table 1:** Various amounts of acids and alcohols added to the artificial esterification system.

Acid and Alcohol (mL)	Treatment				
	1	2	3	4	5
Caproic acid	0	0.1	0.2	0.3	0.4
Lactic acid	0	0.1	0.2	0.3	0.4
Acetic acid	0	0.1	0.2	0.3	0.4
Butyric acid	0	0.1	0.2	0.3	0.4
Ethanol	6	8	10	12	14

**Table 2:** Changes in enzyme activity and esterifying power before and after mutagenesis of *Bacillus velezensis*.

Testing Index	Mutagenized Strain WY4-3	Wild Strain WY4
Amylase activity (U/mL)	41.743 ± 0.140	38.251 ± 0.291
Glucoamylase activity (mg/mL)	1.170 ± 0.065	0.580 ± 0.070
Protease activity (μg/mL)	15.499 ± 0.016	39.335 ± 0.031
Esterifying power (mg/g)	205.660 ± 5.480	30.528 ± 2.060

Data are presented as mean ± SD (n = 3).

## Data Availability

The original contributions presented in this study are included in the article. Further inquiries can be directed to the corresponding authors.
